# LRSCnet: Local Reference Semantic Code learning for breast tumor classification in ultrasound images

**DOI:** 10.1186/s12938-021-00968-3

**Published:** 2021-12-17

**Authors:** Guang Zhang, Yanwei Ren, Xiaoming Xi, Delin Li, Jie Guo, Xiaofeng Li, Cuihuan Tian, Zunyi Xu

**Affiliations:** 1 School of Software, Shandong University, Jinan, China; 2 Health Management, The First Affiliated Hospital of Shangdong First Medical University & Shandong Provincial Qianfoshan Hospital, Jinan, China; 3 School of Computer Science and Technology, Shandong Jianzhu University, Jinan, China; 4 School of Medicine, Shandong Universit, Jinan, China; 5 Health Management Center, QiLu Hospital of Shandong University, Jinan, China

**Keywords:** Ultrasound image classification, Small data, Semantic code learning

## Abstract

**Purpose:**

This study proposed a novel Local Reference Semantic Code (LRSC) network for automatic breast ultrasound image classification with few labeled data.

**Methods:**

In the proposed network, the local structure extractor is firstly developed to learn the local reference which describes common local characteristics of tumors. After that, a two-stage hierarchical encoder is developed to encode the local structures of lesion into the high-level semantic code. Based on the learned semantic code, the self-matching layer is proposed for the final classification.

**Results:**

In the experiment, the proposed method outperformed traditional classification methods and AUC (Area Under Curve), ACC (Accuracy), Sen (Sensitivity), Spec (Specificity), PPV (Positive Predictive Values), and NPV(Negative Predictive Values) are 0.9540, 0.9776, 0.9629, 0.93, 0.9774 and 0.9090, respectively. In addition, the proposed method also improved matching speed.

**Conclusions:**

LRSC-network is proposed for breast ultrasound images classification with few labeled data. In the proposed network, a two-stage hierarchical encoder is introduced to learn high-level semantic code. The learned code contains more effective high-level classification information and is simpler, leading to better generalization ability.

## Background

Breast cancer is the most common malignancy among women in the world. Early diagnosis and treatment can increase the survival rate. Medical image plays an important role for early diagnosis of breast cancer [1, 2]. As one of the most commonly used medical image, ultrasound image is cheaper and safer for patients. In addition, it is more sensitive for abnormalities detection in dense breasts.

However, interpretation of breast ultrasound images (BUS images) depends on radiologists’ expertise, result in subjective diagnosis. In order to tackle this problem, it’s necessary to develop automatic computer-aided diagnosis (CAD) system [3–5]. Generally speaking, the breast ultrasound CAD system mainly includes three stages: lesion segmentation, feature representation and lesion classification.

Feature representation is the essential basis for breast lesion classification. Texture-based features [6–11] are commonly used features for ultrasound image classification because they can represent the scattering properties of breast tissues reasonably. However, they ignore the accurate classification on images from different ultrasound scanners. In order to handle texture variations of BUS images acquired with distinct ultrasound devices, Yang et al. [12] firstly decomposed each BUS image into multiple ranklet images. And then, gray-level co-occurrence matrix (GLCM)-based texture features were extracted from ultrasound images via multi-resolution ranklet transform. Gómez-Flores et al. [13] processed the input image using the ranklet transform, and then extract the auto-mutual information-based texture features from each ranklet image. Ranklet-based features is helpful to improve the robustness. However, Ranklet transformation ignores the local details of the images. In addition, the dimension of Ranklet-based features is high, which may cause curse of dimensionality. In order to tackle these two problems, Xi et al. [14] proposed LSP-Ranklet transformation method by exploring the neighbor information. Based on the LSP-Ranklet-based texture features, multi-task learning is also used to select the robust features. However, it’s difficult to train an effective multi-task learning model by using small data.

In recent years, deep learning has achieved great success in object classification due to its powerful feature learning abiltiy. Huynh et al. [15] extracted features using pre-trained convolutional neural network (CNN) and then used to support vector machine (SVM) classifiers to distinguishing benign and malignant lesions. Xie et al. [16] selected four pre-trained classification network structure, such as LeNet, AlexNet [17], ZFNet and ResNet [18] for automated feature extraction and classification. Alexander Ciritsis et al. [19] use deep convolutional neural network for automatic classification of breast lesions. Razmjooy et al. proposed a new derivative-free meta-heuristic algorithm, which achieved good performance on some optimization problems [20]. Xu et al. proposed an automatic computer-assisted method based on satin bower bird optimization (SBO) algorithm to optimize the rationality of the number of hyperparameters in CNN [21]. The Razmjooy et al. proposed to apply the imperialist competition algorithm to optimize the neuro-fuzzy system and used five new features to train the system, which resulted in a higher classification accuracy [22]. In deep learning framework, sufficient training data with annotation is one of the key factor to improve the performance. However, it’s difficult to collect sufficient ultrasound images with annotation. Moreover, the small data problem may cause reduction of generalization ability of the model. Shi et al. [1] extracted the texture feature firstly, and then developed the stacked deep polynomial network to learn high-level representation for small data classification. However, developing a deep CNN framework may further improve class performance for image classification task because CNN has advantages on effective local information learning than other networks.

**Fig. 1:**
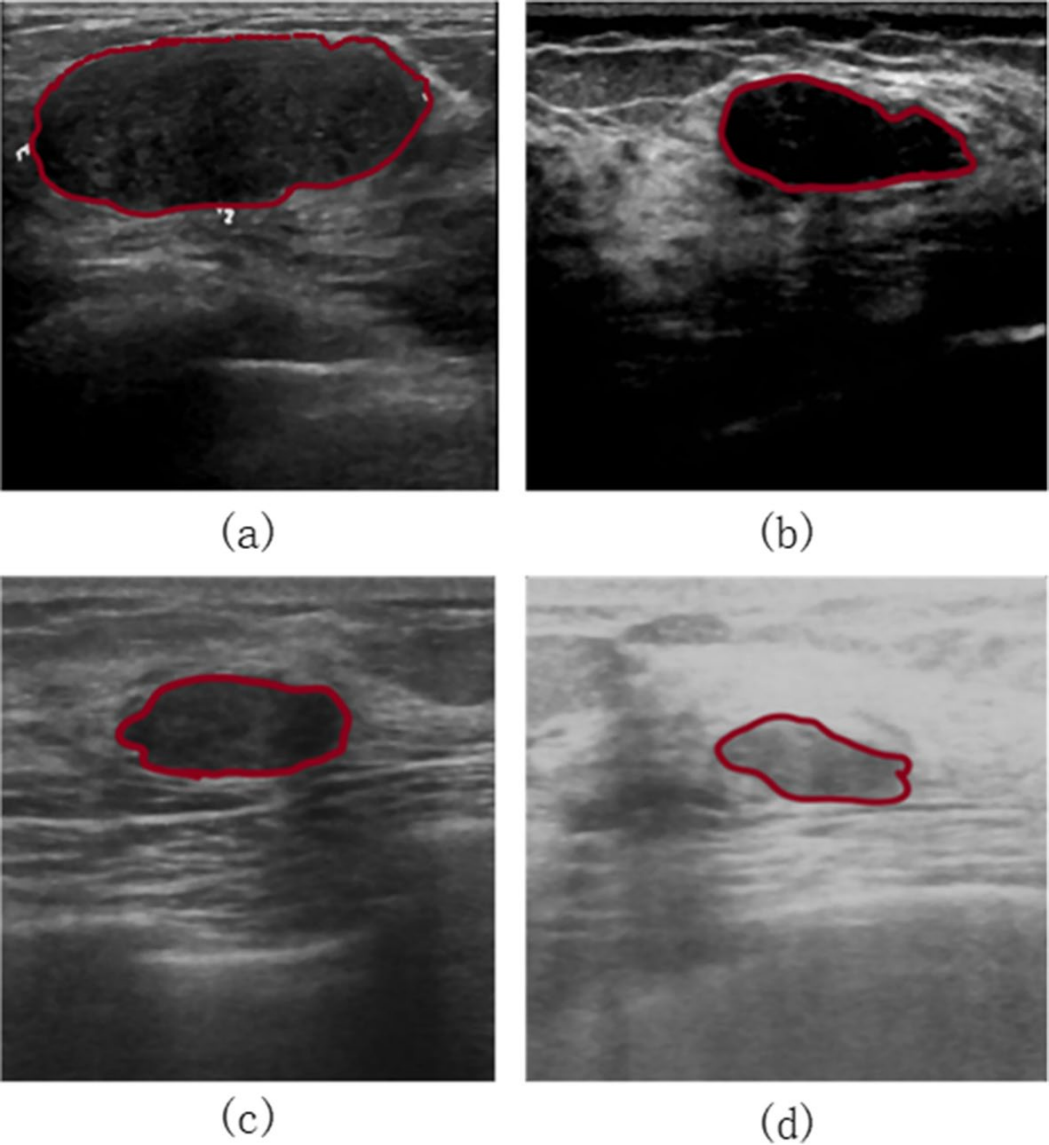
Breast images form four different devices

However, two challenges arise for existing methods. (1) The intra-class variance occurs, result in performance degradation. Considering texture has ability to capture the discriminative scattering properties of breast tissue, some texture-based features have been proposed and achieved satisfactory performance. However, they ignore the accurate classification on images from different ultrasound devices. The texture variance is occurred between the same class due to different imaging principle of ultrasonic devices, result in the large intra-class variance. Figure 1 gives four images from different devices. The four images belong to benign lesion. As shown in this figure, the texture of the second image is smooth while others are coarse, result in the large intra-class variance. (2) It’s difficult to collect sufficient labeled training data, which causes the reduction of generalization ability. Sufficient training data with labels is important to improve the classifier performance. Unfortunately, it’s difficult to collect sufficient ultrasound images with annotation in the real world due to the difficulty of biopsy label collection, limiting performance improvement.

Based on the above idea, this paper develops a novel Local Reference Semantic Code (LRSC) network for lesion classification. In this paper, local reference refers to the common local structure of benign and malignant lesions. Effective local structure is helpful to distinguish class and reduce the intra-class variance [23–28]. The local structure extractor is firstly developed to learn local references via optimizing the developed class guided distance-based object function. The generated local references contain class information. After that, a two-stage hierarchical encoder is developed to encode the local structures of lesion into the high-level semantic code. The proposed encoder contains similarity learning module and semantic transfer module. In the first stage, the encoder learns the local similarity code via the similarity learning module. The similarity learning module is developed based on a deep learner which can learn effective relation between the local patch and local references. In the second stage, based on the learned similarity code, the encoder further generated the semantic code via semantic transfer module. The obtained semantic code can be seen as the high-level class description. It has ability to measure the relation between the whole image and certain common local characteristics of tumors. Therefore, the global code is robust to intra-class variance. In addition, it contains more effective high-level classification information and is simpler, leading to better generalization ability. At last, the self-matching layer is proposed to obtain the final classification result, eliminating time-consuming traditional distance matching, improving the matching speed. The experimental results on our database demonstrate the effectiveness and robustness of the proposed method. The main contribution of this paper can be summarized as follows:

1. In order to solve the problem of intra-class variance and small data, a novel LRSCnet is proposed. On one hand, the common local structure is learned to tackle the problem of intra-class variance. On the other hand, a novel high-level semantic feature is learned to avoid overfitting.
2. Local reference is proposed to describe the common local characteristics of lession. The local structure extractor is developed to learn local reference in the proposed network.
3. In order to encode the local structures of lesion into semantic code, a novel two-stage encoder which contains similarity learning module and semantic transfer module is proposed.
4. The self-matching layer is introduced for final matching, eliminating traditional time-consuming distance matching and classifier, improving the matching speed.

This paper is organized as follows. The results are given in "Results" section. The discussion based on the experimental results is give in "Discussion" section. This paper is summarized in "Conclusion" section. The proposed method is given in "Method" section.

## Results

### Data description

To verify the effectiveness of the proposed method, experiments are conducted on our breast ultrasound images dataset [14]. The BUS images database contains 186 cases which are collected from 135 benign cases and 51 malignant cases provided by the Qianfushan Hospital of Shandong Province. The images were captured from four ultrasonic devices whose types are ALOKA $\alpha$ 10, AplioXG, GE LOGIQ E7 and SIEMENS Sequoia 512 respectively, as shown in Table 1. In addition, lesion boundaries were manually demarcated by experienced radiologists, and lesion ROI can be selected according to the lesion boundary labeled.

**Table 1:** Breast images from different devices

Finding	ALOKA $\alpha$ 10	AplioXG	GE LOGIQ E7	SIEMENS Sequoia 512
	Number of Cases	Number of Cases	Number of Cases	Number of Cases
Benign	47	38	47	3
Malignant	14	6	28	3
Total	61	44	75	6

### Experience setting

In our experiment, the values of learning rate, momentum and weight decay are setting to 0.001, 0.9 and $10^{-6}$ respectively. Eighty percent of benign lesions and malignant lesions are selected as the training data, and remain images are regarded as the testing data. In addition, test metrics are used such as area under the ROC curve (AUC), accuracy (ACC), sensitivity (Sen), specificity (Spec), positive and negative predictive values (PPV and NPV) [12] to evaluate the diagnostic performance of the proposed method. Specifically, each index of every mammography-ultrasound pair is defined as:

$$\begin{aligned} \text{Accuracy}= & {} \frac{TP+TN}{TP+TN+FP+FN},\\\ \text{Sensitivity}= & {} \frac{TP}{TP+FN},\\ \text{Specificity}= & {} \frac{TN}{TN+FP},\\ \text{ PPV}= & {} \frac{TP}{TP+FP},\\ \text{NPV}= & {} \frac{TN}{TN+FN} \end{aligned}$$

where TP, TN, FP and FN are shown in Table 2.

**Table 2:** Illustration of TP, TN, FP and FN

	Positive (benign)	Negative (malignant)
True	TP	FP
False	FN	TN

### Experimental results

**Fig. 2:**
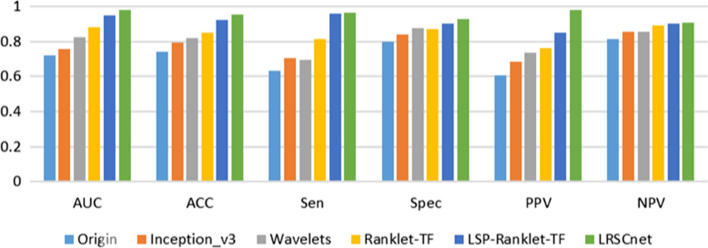
Classification results of different methods on lesion images

Firstly, the performance of LRSCnet with the textural features extracted by wavelets, Ranklet-TF [12], LSP-Ranklet-TF-MTL [14] and the Google inception_v3 model [29] are compared. The inception_v3 model was initialized by the ImageNet pre-training model and fine-tuned on our dataset. In addition, data augmentation was also used in the inception_v3 model training process. The experimental results of the different methods are shown in Fig. 2.

As shown in this figure, it can be observed that LRSCnet achieves the best performance in each of the evaluated metrics, AUC, ACC, Sen, Spec, PPV, and NPV are 0.9540, 0.9776, 0.9629, 0.93, 0.9774 and 0.9090, respectively.

**Fig. 3:**
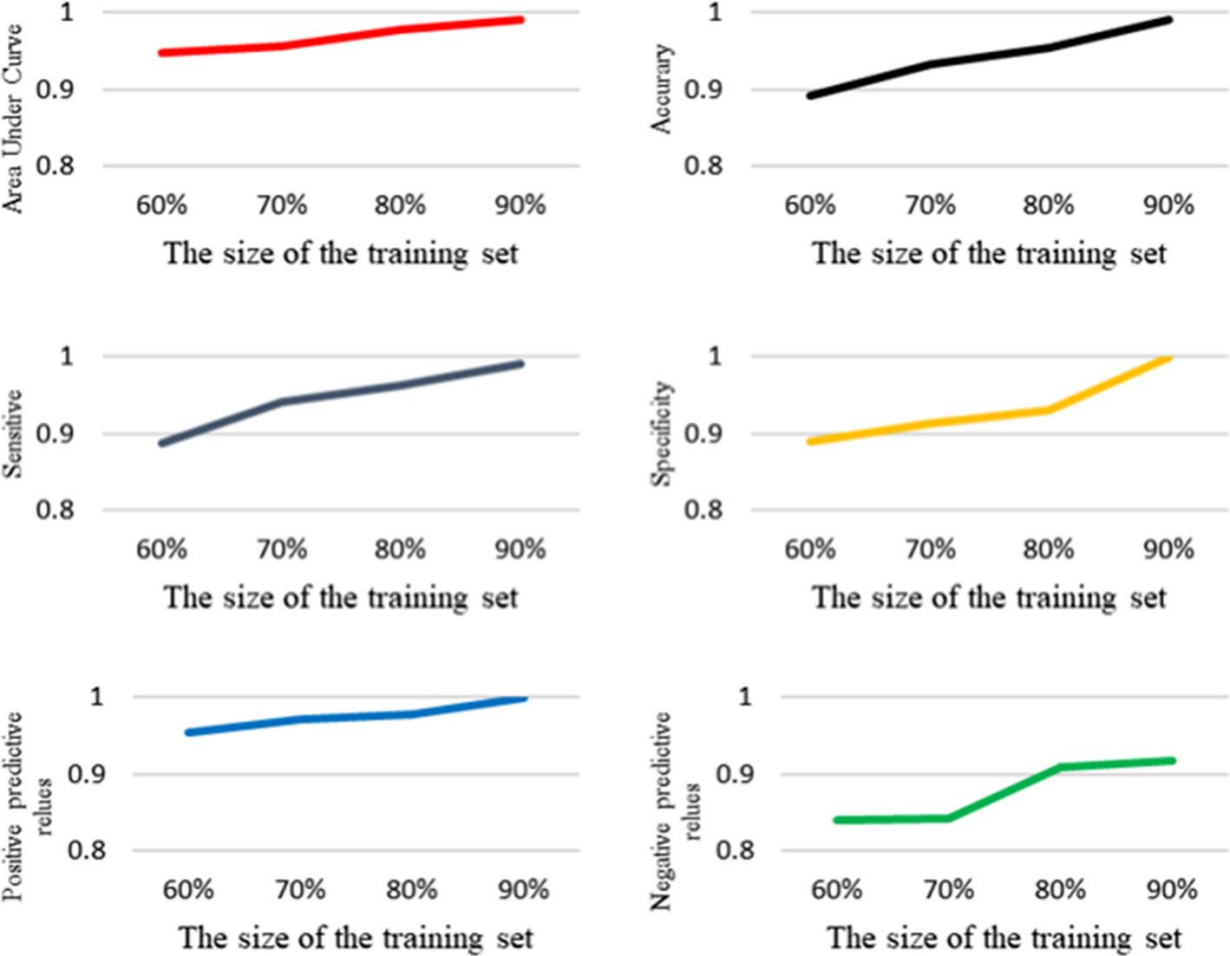
Classification results of different data size on lesion images

The results on different training sets whose size are 60%, 70%, 80% and 90% of the original dataset are reported respectively, shown in Fig. 3. As shown in this figure, with the number of train data increasing, LRSCnet has achieved better performance. LRSCnet can outperform traditional methods by using only 70% training data.

**Table 3:** Computation time of LSP-Ranklet-TF-MTL and LRSCnet

Method	Time (ms)
LSP-Ranklet-TF-MTL	0.012
LRSCnet	0.009

Table 3 lists computation time of LSP-Ranklet-TF-MTL and LRSCnet. As show in this table, LRSCnet spent less match time because the obtained similarity code is short and contains class information. The self-matching layer can generate classification result via statistical analysis of each bit, eliminating time-consuming classification, distance calculation, improving matching speed.

## Discussion

The proposed method improves the effectiveness and efficiency due to its several advantages:

1. Local reference is proposed to describe the tumor’ common local characteristics which is robust to local intra-class variance. In addition, a novel two-stage encoder is developed to encode the local structures of lesion into shot high level semantic code which has ability to overcome the over-fitting problem.
2. The self-matching layer is introduced for final matching, eliminating traditional time-consuming distance matching and classifier, improving the matching speed.

The experimental results demonstrate the effectiveness of the proposed method. As shown in Fig. 2, compared with state-of-the-art classification algorithms, Accuracy, AUC, Sen, Spec, PPV, and NPV have improved respectively. LSP-Ranklet-TF achieves the better performance among the features extracted by shallow model. The proposed LRSCnet outperforms LSP-Ranklet-TF. Multi-task learning model can select robust information from the complex LSP-Ranklet-based features. However, it’s difficult to train an effective multi-task learning model by using small data. On the contrast, LRSCnet has ability to learn high-level class description. The learned semantic code contains more knowledge about lesions and is simplier, which is helpful to improve the generalization ability. Compared with deep learning method, LRSCnet also outperforms inception_v3 model. Compared with traditional deep learning method, LRSCnet focus on effective learning of local structure which is robust to intra-class variance.

As shown in Fig. 3, LRSCnet can learn similarity code which represents the relation between the target class and common local structure, containing more knowledge of the lesions. In addition, the learned code is shot. Therefore, LRSCnet has better generalization ability which is helpful to tackle the problem of small data.

**Fig. 4:**
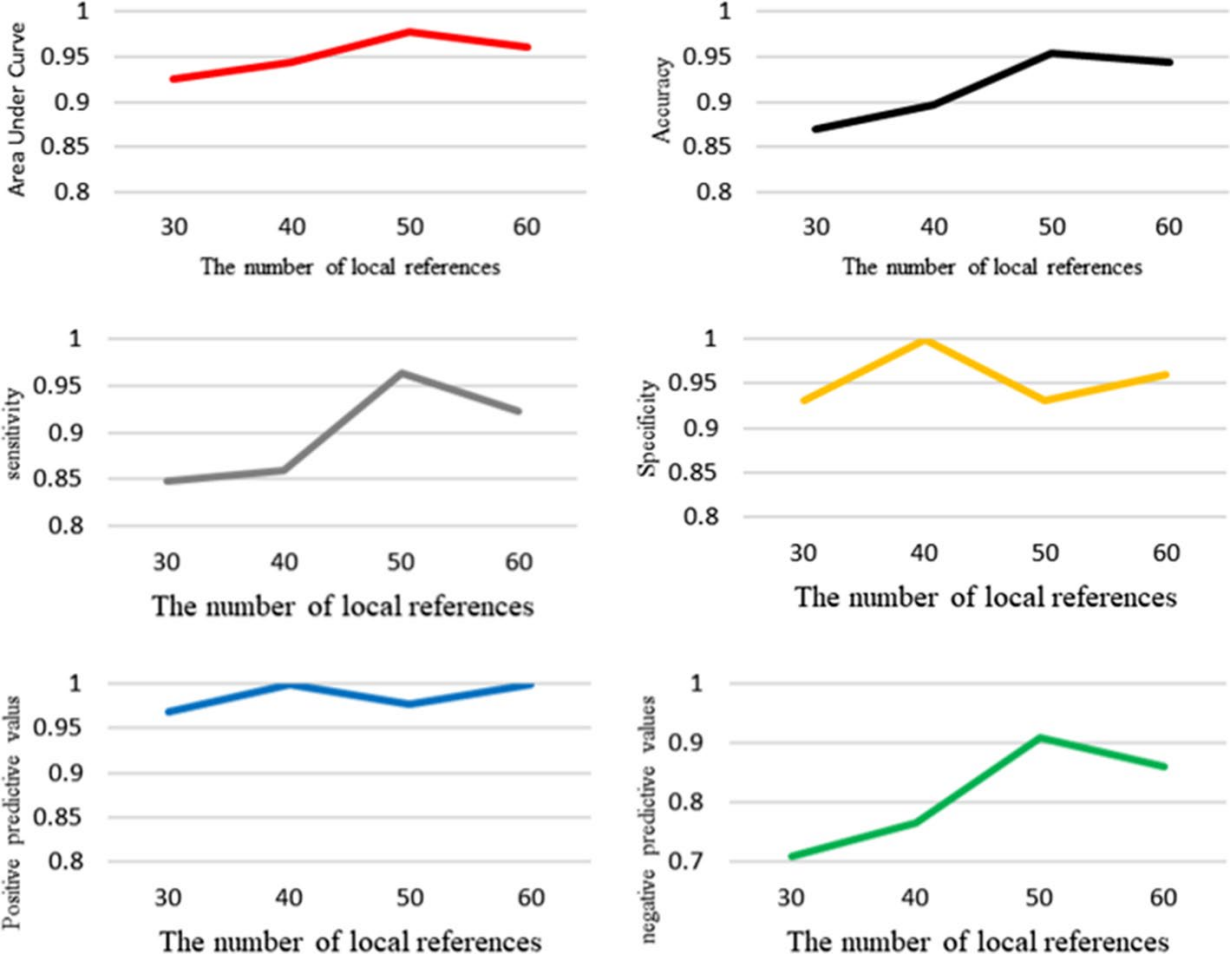
The effect of the number of local references on the performance of LRSCnet

The performance of LRSnet was tested with different number of local references. The number is set as 30,40,50,60 and 70 respectively, shown in Fig. 4. It was observed that with the number of local references increasing, the performance is improved. However, when the number is larger than 50, the performance is degraded. The reason may be that more local details can be learned with the number of local references increasing. However, noise may be introduced when the number of local references is too large, result in the performance degradation.

The main disadvantage of this study is that it has more hyper-parameters. Compared with traditional neural network, the extra hyper parameter for local references is introduced. In this paper, the number of local references are given via experimental results. However, the number of references has affected the classification performance. Therefore, develop a new method to automatically achieve the optimal parameters for local references is a direction of the future work.

## Conclusion

This paper proposes LRSC-network for breast lesion classification with few labeled data. In the proposed network, the local structure extractor is firstly developed to learn the local reference which is robust to large intra-class variance. Moreover, a two-stage hierarchical encoder is introduced to learn high-level semantic code. The learned code contains more effective high-level classification information and is simpler, leading to better generalization ability. The experimental results on our database demonstrate the effectiveness of the proposed method. AUC, ACC, Sen, Spec are 0.9540, 0.9776, 0.9629, 0.93, respectively. In the future work, we will extend the proposed method to the semi-supervised learning framework.

## Method

**Fig. 5:**
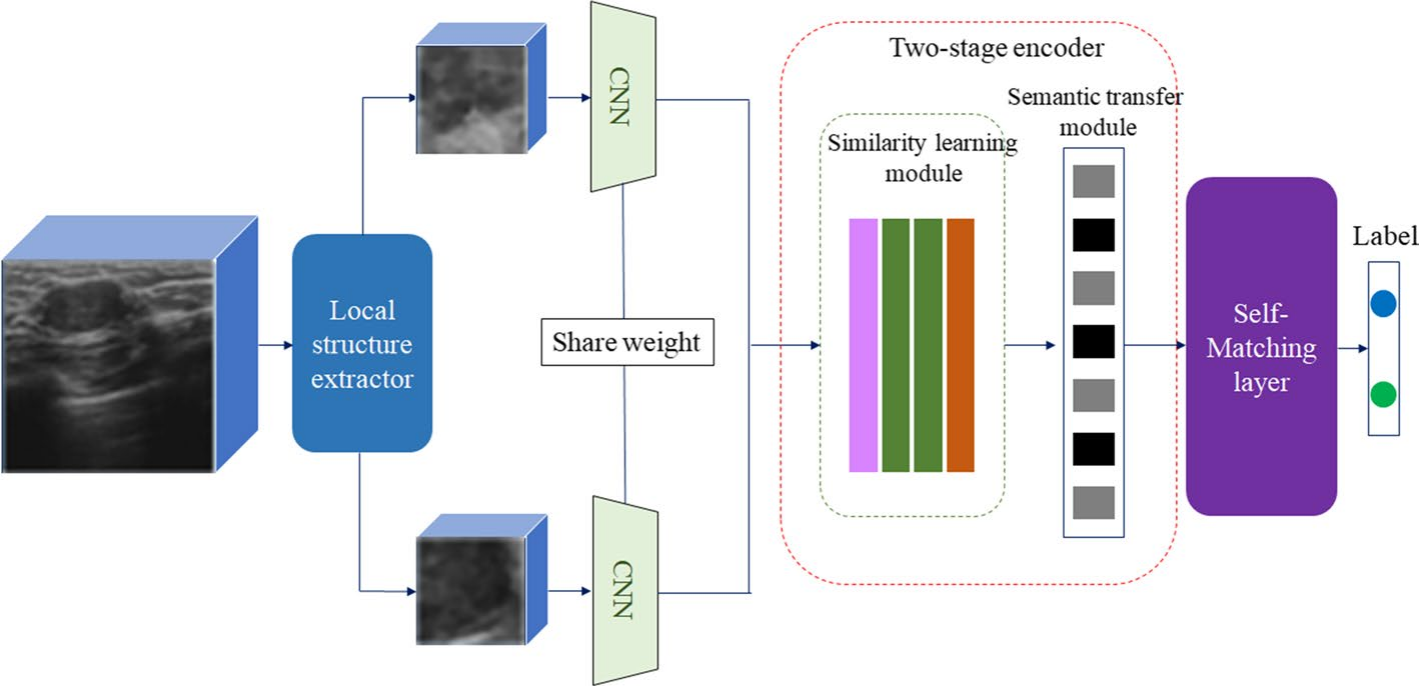
The architecture of LRSCnet

This paper proposes a novel Local Reference Semantic Code (LRSC) network for breast lesion classification. Figure 5 shows the architecture of the LRSCnet. In the proposed network, the local structure extractor is firstly developed for local references learning and local patches generation. Then two pipeline CNN feature extractors are used to learn the features of the acquired local patch and local reference. After that, the two-stage encoder which contains similarity learning module and semantic transfer module is introduced to learn the global semantic code. The learned code has ability to measure the similarity among the input image and local references. Finally, the self-matching layer is developed for faster classification.

### Convolutional Neural Network (CNN)

In recent years, CNN has achieved significant improvement in image processing area. Generally speaking, traditional CNN are composed of three types of layers: convolutional layers,pooling layers and fully connected layers. The convolution layer is composed of different convolution banks. They firstly perform convolution of the local patch of input. After that, corresponding convolutions are summed up, and then passed through a nonlinear activation function such as a ReLU to generate different feature maps which can capture local statistics of images. The pooling layer aims to reduce the complexity of the features and retain the important information which is robust to rotation. Max pooling is common pooling method. It computes the maximum of a local patch of units in one feature map. The Fully Connected layer is used to combines learned local features into global features.

### Local structure extractor

Local information plays an important role in the breast ultrasound images classification [8]. Moreover, capturing effective local structure is helpful to distinguish class and reduce the intra-class variance [6, 23]. Based on this idea, local structure extractor is developed to capture the effective local information of the lesions.

In the proposed network, local structure extractor aims to learn local references and generate the local patches. Local references are learned by optimizing the class guided distance-based objective function. In addition, the local patches are generated by using a slide window of size m × m.

According to the definition of local reference in this paper, local reference should have following two properties: (1) It represents common local characteristics of the lesion, such as, internal echo, posterior acoustic behavior [8, 30] and so on. (2) It contains class information, i.e. the local reference belongs to benign or malignant lesions.

According to the two properties, it can be inferred that the local reference can be seen as the local structure atoms with class information. For example, the local patches extracted from the posterior echo of benign lesion should be similar with local reference which describes the posterior echo of the benign lesion. Based on the above idea, the local reference is learned by minimizing the class guided distance-based objective function as follows:

1 $$\begin{aligned} \underset{\mu \ \ \vartheta }{min}\left( 1-p\left( x \right) \right) \sum _{i=1}^{k}\sum _{x\in M_{I}}\left\| x-\mu _{i} \right\| _{2}^{2}+p\left( z \right) \sum _{i=1}^{r}\sum _{z\in B_{i}}\left\| z-\vartheta _{i} \right\| _{2}^{2} \end{aligned}$$

In Eq. (1) [31], variable x and z denotes the local patch extracted from the benign lesion and the malignant lesion respectively, p(t) is the indicate function whose value is 1 or 0. If input t belongs to the benign lesion, its value is 0, and vice versa. k and r denote the number of local references from benign lesion and malignant lesion respectively. $\mu _i$ and $\vartheta _i$ denotes the i-th local reference of benign lesion and malignant lesion respectively.

The class guided distance-based objective function minimization can be achieved by the iterative optimization: (1) the initial value of the local references is given randomly. (2) similarity calculation. Euclidean distance is used to measure the similarity between the local reference and the local patch.$M_i$ denotes the local patch set which is more similar with the i-th local reference from benign lesion while $B_i$ denotes the local patch set which is more similar with the i-th local reference from malignant lesion.(3) local references updating. After obtaining the local patch set M and B, the local references are updated as Eq. (2) [31] and Eq. (3) [31]:

2 $$\begin{aligned} \mu _{i}= & {} \frac{1}{\left| M_{i} \right| }\sum _{x\in M_{i}}x \end{aligned}$$

3 $$\begin{aligned} \vartheta _{i}= & {} \frac{1}{\left| B_{i} \right| }\sum _{z\in B_{i}}z \end{aligned}$$

The step of similarity calculation and local references updating are repeated. The iteration is stopped when the value of the local reference is not updated, and the final local references can be obtained.

### Feature extraction

**Fig. 6:**
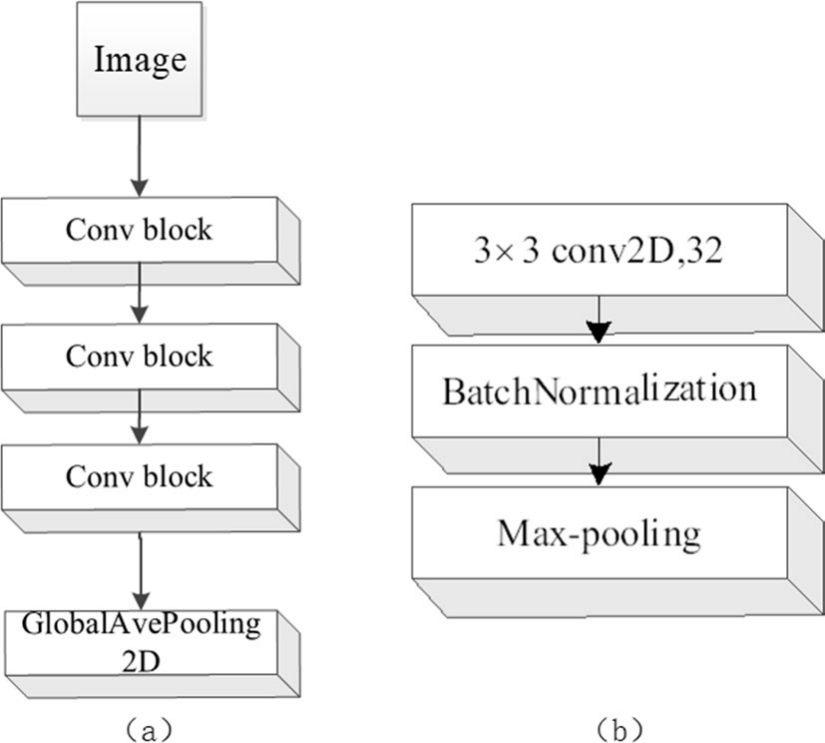
Network structure of the CNN feature extractor. **a** Network structure of feature extractor, **b** conv block

The flexible two pipeline network architecture which shares the same architecture and tied parameters is used for feature extraction. In the proposed network, the CNN pipeline is composed of three convolutional block, each of which is a 3 $\times$ 3 convolution with 32 filters followed by batch normalization, ReLU non-linearity and 2$\times$2 max-pooling. At the end of the last dense block, a global average pooling is performed, as shown in Fig. 6, (a) is the network structure of the CNN feature extractor, and (b) is the network structure of the Conv block in (a).

### Two-stage Encoder

In the proposed network, the two-stage encoder is proposed to encode the local structure of lesion into the high-level semantic code. It mainly contains similarity learning module and semantic transfer module. Similarity learning module is developed to learn the similarity between local references and local patches. Based on the obtained local similarity feature, semantic transfer module is developed to generate the semantic code which measures the relation between the image and the local references with classification information.

#### Local similarity learning

In the first-stage encoder, similarity learning module is developed to learn the similarity between local references and local patches. The module is constructed by using a neural network which contains three layers. The first two layers are used to further learning effective feature while the last layer is used to learn the similarity between local references and local patches.

The input of the similarity learning module is a vector, thus the extracted CNN features of these two samples should be combined into one feature vector. Inspired by learning to rank framework [32], the similarities between the two samples are compared by combining two features with subtracted operation, as show in Eq. (4).

4 $$\begin{aligned} v_{i}\left( x,l \right) =\left| f_{\varphi }\left( x_{i} \right) -f_{\varphi }\left( l_{i} \right) \right| \end{aligned}$$

In Eq. (4), $f_{\varphi }$ denote the feature extractor, $v_i$ denotes the combined feature, $x_i$ is local patch, $l_i$ is local reference. Compared with traditional concat methods, the proposed method can reduce the dimension of the combined features.

The first two layers which are fully connected layer further learn effective features via hierarchical nonlinear feature transformation, as shown in following equation:

5 $$\begin{aligned}&F= h\left( h\left( v\left( x_{i},l_{i} \right) \right) \right) \end{aligned}$$

6 $$\begin{aligned}&h\left( v \right) = relu\left( Wv+b \right) \end{aligned}$$

where v ($x_i$,$l_i$) is the combined feature, F is the learned feature, W and b are the parameters to be learned.

Based on the learned features, the similarity between local patch and local reference can be learned by using the last layer, as shown in Eq. (7).

7 $$\begin{aligned} s=softmax\left( F \right) \end{aligned}$$

where is the similarity between the local patch and the local reference. If the local patch and the local reference are related, the similarity between them is higher.

Therefore, for arbitrary local patch p, its local similarity feature $F_{ls}$ can be learned. The i-th element in $F_{ls}$ denotes the similarity between p and i-th local reference. The length of the feature vector $F_{ls}$ is the number of the local references. For example, the number of local references is 100, the dimension of $F_{ls}$ is 100. Since the local reference contains class information, the learned feature also contains certain class information.

#### Semantic code generation

**Fig. 7:**
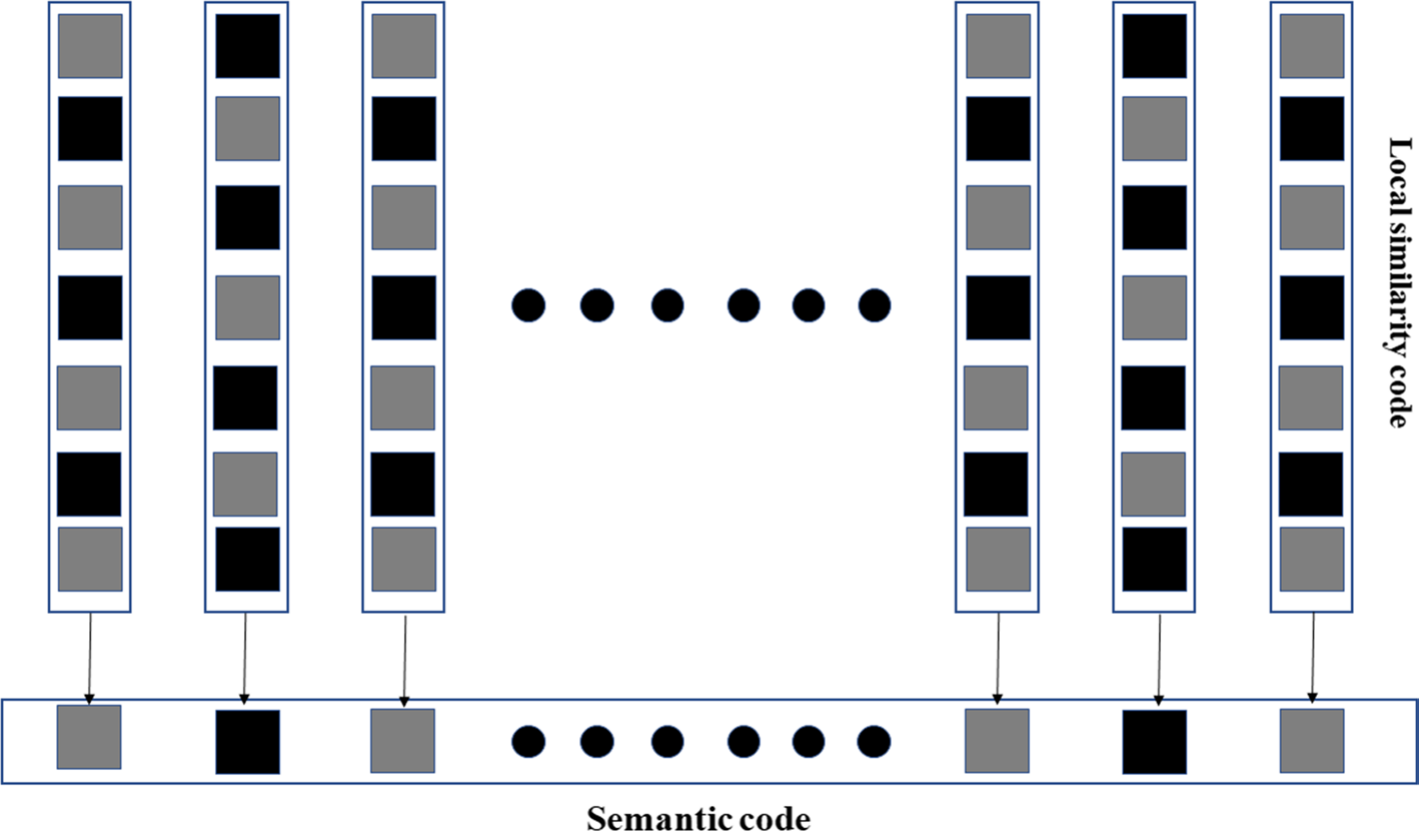
Semantic code generation

The semantic transfer module is developed to transform the obtained local similarity codes into the high-level semantic code, show in Fig. 7.

The semantic code is composed of semantic bits, arbitrary semantic bit is obtained based on certain local similarity code, as shown in following equations. In Eq. (8), $K^1$ is the number of local reference, f is the local similarity feature, $f_k$ denotes the k-th element of the feature. *B* is the semantic bit. In this paper, if the value of *B* is 1, it represents that the local patch belongs to benign lesion, and vice versa.

8 $$\begin{aligned}&p=\frac{\sum _{k=1}^{K^{1}}f_{k}}{K^{1}} \end{aligned}$$

9 $$\begin{aligned}&B\left( p \right) = \left\{ \begin{matrix} 0, \quad &{} p< 0.5\\ 1, \quad &{} p\ge 0.5 \end{matrix}\right. \end{aligned}$$

10 $$\begin{aligned}&Sc\left( I \right) =\left( B\left( p_{_{1}} \right) ,B\left( p_{2} \right) ,\cdots ,B\left( p_{n} \right) \right) \end{aligned}$$

The generated semantic code is the high-level class description of the image *I*. Each bit of the code has ability to measure the relation between the lesion and the certain local structure. Therefore, the generated code can describe more knowledge of lesion by using all bits, which is more robust to noise bit caused by intra-class variance. In addition, each bit contains high-level class information. Moreover, the learned similarity code is simpler, which is helpful to improve the generalization ability.

The self-matching layer is introduced for the final lesion classification. Considering that the semantic code contains the class information, the final classification result can be obtained via simple statistical analysis of each bit, shown in Eq. (11). In this equation, n is the number of dimension of generated semantic code for the given image. $B_k$ is the k-th bit in the global semantic code. 0.5 is set as the threshold. If *l* is larger than 0.5, the image belongs to benign lesion, and vice versa.

11 $$\begin{aligned} l= \frac{\sum _{k=1}^{n}B_{k}}{n} \end{aligned}$$

Compared with traditional classification method, self-matching layer can generate the classification result without training, eliminates traditional time-consuming classification method, such as classifier training, distance calculation, improving matching speed.

## Data Availability

The datasets used and/or analyzed during the current study are available from the corresponding author on reasonable request.

## Abbreviations

LRSC: Local Reference Semantic Code
BUS: Breast ultrasound images
CAD: Computer-aided diagnosis
GLCM: Gray-level co-occurrence matrix
CNN: Convolutional neural network
SVM: Support vector machine
SBO: Satin bower bird optimization
ROI: Region of interest
AUC: Area under curve
ACC: Accuracy
Sen: Sensitivity
Spec: Specificity
PPV: Positive predictive values
NPV: Negative predictive values
